# Perceptions of Sexual Healthcare Provision in Tanzania: a Key Informant Qualitative Study

**DOI:** 10.1007/s13178-021-00607-5

**Published:** 2021-07-03

**Authors:** Lucy R. Mgopa, Michael W. Ross, Gift Gadiel Lukumay, Stella Emmanuel Mushy, Ever Mkony, Agnes F. Massae, Dorkas L. Mwakawanga, Sebalda Leshabari, Inari Mohamed, Maria Trent, James Wadley, Zobeida E. Bonilla, B. R. Simon Rosser

**Affiliations:** 1 Department of Psychiatry and Mental Health, School of Medicine, Muhimbili University of Health and Allied Sciences (MUHAS), Dar es Salaam, Tanzania; 2 Program in Human Sexuality, Department of Family Medicine, University of Minnesota, Minneapolis, MN, USA; 3 Department of Community Health Nursing, School of Nursing, Muhimbili University of Health and Allied Sciences (MUHAS), Dar es Salaam, Tanzania; 4 Department of Epidemiology and Community Health, School of Public Health, University of Minnesota, Minneapolis, MN, USA; 5 Department of Adolescent and Young Adult Medicine, Johns Hopkins University Schools of Medicine and Public Health, Baltimore, MD, USA; 6 Department of Counselling and Health Services, Lincoln University, Philadelphia, PA, USA

**Keywords:** Culture, Sexual health, Africa, Youth, HIV

## Abstract

**Introduction:**

Sexual health care services must be standard and unbiased, guided by a structured health care system. There is a scarcity of data on how sexual health care is delivered in Tanzania.

**Methods:**

To address this gap, in July 2019 we interviewed eleven key informants: cultural and public health experts, and political, religious, and community leaders, selected from different organizations in Dar es Salaam, Tanzania. Participants were asked for their opinions about clinical practices of health care professionals when providing care to patients, with an emphasis on sexual health.

**Results:**

Participants’ responses were classified into three subcategories: strengths, barriers, and gaps in sexual health care. Availability of services, service delivery to adults, and code of conduct were among the strengths observed in clinical care services. Barriers included the health care provider’s attitudes, moral values, and inadequacy in health policies and treatment guidelines. Vulnerable populations including youth were frequently reported to face most challenges when seeking sexual health care services. In terms of gaps, informants emphasized gender equity in sexual health services provision within care settings.

**Conclusion and Implication:**

Data indicate that lack of training in sexual health and guidelines for dealing with sexual issues are a barrier to comprehensive health care. These findings can inform the main areas for curriculum developers to focus on, when developing an Afro-centric sexual health curriculum suitable for students in health care professional courses. Moreover, these findings can be useful when developing treatment guidelines and policies that are beneficial to the sexual health wellbeing of individuals.

## Introduction

Health care services incorporate those delivered to target physical, mental, emotional, and sexual wellbeing and health. Sexual health covers a broad range of problems from HIV/AIDS, sexually transmitted illnesses (STIs), and unwanted pregnancies, to sexual dysfunctions, sexual violence as well as sexual orientation, gender, and sex education concerns (WHO, 2017). The delivery of health care services including those targeting sexual health should be comprehensive, accessible, patient focused, non-discriminatory, evidence-based, confidential, managed with trained staff, and governed with a good leadership (FSRH, 2013; WHO, 2018).

In Tanzania, the provision of sexual health care is focused on HIV and STI services, sexual and reproductive health care such as use of contraceptives, youth pregnancies and post abortion care, management and prevention of female genital mutilation/cutting (FGM/C), treatment of sexual dysfunction, management of sexual violence and sexual-related problems among key populations including sex workers (SW) and men who have sex with men (MSM), and provision of non-formal sex education (Cardoso & Mwolo, 2017; Ministry of Health and Social Welfare (MoHSW) [Tanzania Mainland], Ministry of Health (MoH) [Zanzibar], (NBS), (OCGS), & (ICF), 2015; Marcusán et al., 2015; Ministry of Health and Social Welfare (MoHSW) & (NACP)[Tanzania], 2014; Ministry of Health and Social Welfare (MoHSW) & Ministry of Home Affairs (MoH)[Tanzania], 2015; Ministry of Health, Community Development, Gender, Elderly and Children (MoHCDGEC)[Tanzania], 2017). Countries in Sub-Saharan Africa are faced with multiple challenges in their health care systems that act as barriers to provision of health care, and in particular to the delivery of sexual health care (Akatukwasa et al., 2019; Jonas et al., 2018; Oleribe et al., 2019). Given these different sexual health challenges that are prevalent in Africa (Ameyaw et al., 2019; Hokororo et al., 2015; Pallangyo et al., 2016; UNICEF, 2019; Yovwin et al., 2015; Yusuf & Fessha, 2013), Tanzania also faces similar impediments as many other African countries when providing sexual health care services to target populations, despite the integration of sexual health care in some of the local health facilities (Mbeba et al., 2012; Mchome et al., 2015; Ministry of Health and Social Welfare (MoHSW) & Ministry of Home Affairs (MoH) [Tanzania], 2015; Wojcicki, 2017).

Frequently, there are a set of structural and logistical challenges to providing good sexual health care across Africa. These include lack of private rooms to ensure confidentiality and privacy, time constraints including lack of scheduling for attending patients, high volume of patients, and unavailability of specialized sexual health services for referral. Even in situations where some of these are present, then cost becomes a major barrier to most patients accessing services (Mbeba et al., 2012; Mchome et al., 2015; Wojcicki, 2017). In low- and middle-income countries, these challenges appear to be evident in both private and public health care facilities; however, improper time schedules, substandard technology, and service delivery as well as client dissatisfaction are higher among public health care settings (Basu et al., 2012; Hutchinson et al., 2011).

At the individual level, health care providers (HCPs) are expected to perform their duties with utmost respect and care to the patients, identify and acknowledge patients’ values, needs, and preferences, and be able to communicate and educate patients well, while promoting healthy lifestyles (Institute of Medicine Committee on the Health Professions Education, 2003). Regarding knowledge about sexual health, HCPs should be well trained and competent with skills to perform well, be culturally sensitive when caring for patients, and have the ability to provide user-friendly services with a positive attitude to recipients of different cultures, ages, or sexual orientation (Fennell & Grant, 2019; Jonas et al., 2017; Lacey, 2017; Ross et al., 2015; WHO, 2010). Without training, HCPs may lack the skills needed to address their patients’ concerns. Negative attitudes towards patients, stigma, hostile reception, use of condemnatory language or communication barriers, and lack of significant knowledge about sexual health are common barriers that have been reported (Duby et al., 2018; Magesa et al., 2014; Mbeba et al., 2012; Mchome et al., 2015; Wanyenze et al., 2017).

Sexual health education is important, and attaining prior knowledge may contribute to improved confidence and attitudes towards handling sexual health problems; this statement was supported in 2016 when Ross and colleagues evaluated a new assessment instrument: the sexual health education for professionals’ scale in Tanzania. This tool was used to assess a change in sexual health confidence, knowledge, and attitudes among nursing and midwifery students following a 2-day interventional workshop. The aim of this evaluation was to tailor and test the assessment tool in an African setting and culture. The results revealed a significant change from pre- to post-test knowledge and confidence on topics regarding sexual health (Ross et al., 2018), showing the effectiveness of the workshop that was conducted, the efficacy of the tool used, and the impact it made to the participating students.

Little is known on how sexual health care is delivered by HCPs in Tanzania. In this current paper, researchers aimed at assessing sexual health practices by health care professionals (providers) in health care settings across Dar es Salaam, Tanzania. The focus is on how well these services are provided including the behavior and engagement of the provider, what is missing, and the difficulties encountered during clinical care provision when attending to sexual health patients. The information obtained will be integrated during the development of a sexual health curriculum that will be used to inform health students in clinical practices in Tanzania, and later on in Sub-Saharan Africa.

## Methods

### Study Design

This study utilized a qualitative interview method as part of a larger mixed-method (qualitative-quantitative exploratory design) formative research for the Training for Health Professionals (THP) study. The purpose of this study was to inform the development of a sexual health teaching curriculum intended for undergraduate clinical students at Muhimbili University of Health and Allied Sciences (MUHAS). This paper utilized key informant interviews from religious, cultural, public health, political, and community organizations. The main aim of this paper was to assess sexual health practices by health care professionals (providers) in Tanzania. The study was a collaboration between MUHAS in Tanzania and the University of Minnesota in the USA. Ethical approval was obtained from the institutional review boards of both universities and the Tanzania National Institute of Medical Research (NIMR).

### Study Participants and Recruitment

Key informants were selected through purposive sampling from July to August, 2019, in Dar es Salaam (Tongco, 2007). Informants were selected based on their roles in the community, first-hand knowledge, experience, and willingness. Those who voluntarily agreed to participate were recruited into the study without coercion. There were eleven key informants, three females and eight males. Participants’ ages ranged between 40 and 76 years, while their years of work experience were between 8 and 43 years. The participants comprised of three religious’ leaders, two cultural experts, three public health experts, two non-government organization (NGO-community) leaders, and one politician who was a representative from the Ministry of Health. The religious leaders were recruited from two Christian denominations—Lutheran and Seventh day Adventist—and there was one Muslim Sheikh. Participants from public health were specialized in community health, epidemiology, and biostatistics, while cultural experts were informants whose educational background and area of specialty were on cultural anthropology and social sciences. Key informants selected from NGOs were specialized in areas of HIV/AIDS care provision, family planning, and research among key populations.

This diversity in expertise allowed us to discern different attitudes, experiences, views, and practices towards provision of sexual health care in Tanzania. The key informants were recruited through a phone call by a member of the research team. After the aims of the interview were explained, verbal consent was requested to interview, and a time and place for the interview identified. During the interview, the eleven participants were likely to speak for themselves, based on their expertise and for the people they serve. Informants did not represent the whole population of Tanzania that has 120 tribes, filled with communities of different social, cultural, and economic characteristics.

### Study Procedures and Data Collection

The interviews were conducted at the informant’s personal office, or somewhere accessible and private chosen by the participant. The languages used were Kiswahili and English with participants choosing which language they preferred to be interviewed in. At the beginning of the interview, participants were handed a short demographic form and a written consent form. Informants were reminded both during the initial phone call and prior to the start of the interview, that their participation was voluntary, confidential, that they could withdraw at any time, and that the interview was being audio-recorded. At the end of the interview, key informants received TZS 120,000 (about US$52) as a token of compensation for transport and meals.

The interview schedule of questions was prepared and reviewed by the whole research team before its administration. While the format and overall content of questions were similar across key informants, each interview guide was tailored slightly to address the informants’ areas of expertise. Topics covered in the interview guide included identification of the most important sexual health concerns in Tanzania, sexual health policies, perception about health professional practices, unmet needs and priorities regarding sexual health, sensitivity of sexual health in Tanzania communities, and questions asking their personal contribution into the development of the African-centric sexual health curriculum. This particular paper focuses on the perception of the key informants regarding health care professionals’ clinical practices; this entailed the strength and weakness/gaps when providing clinical care to the individuals in need of health care, particularly sexual health care.

### Data Management and Analysis

At the end of each interview, all digital audiotape files were labeled with a unique identification number, and uploaded onto a secure password–protected server. The written notes of the interviewer were also typed up and uploaded electronically. The audio files were then transcribed and translated into English and analyzed. Principles of thematic analysis were applied, to identify, organize, and describe the findings of this study (Braun & Clarke, 2006). Coding of the transcripts involved a team-based approach (MacQueen et al., 1998; Richards & Hemphill, 2018) that was implemented throughout all stages of data collection and analysis including codebook development and coding. The team followed a three-step coding process (Saldaña, 2013), beginning with the first step of open coding. This step involved open coding of the transcripts without a predetermined set of codes or theoretical framework. The second step, or first full coding cycle, involved the application of the entire codebook to the transcripts to identify and refine themes. The third step involved a second coding cycle where categories were generated to cluster themes and text with similar meaning. This procedure allowed the team to process, reflect, and debate on the data in order to get an understanding of the experiences between different stakeholders.

The data collection team comprised of six people, and it included faculty from the health professions at MUHAS who are currently practicing in their respective fields, i.e., nurses, midwives, medical doctor, and a PhD student from the University of Minnesota who speaks Kiswahili and English as first languages. The analysis was performed by seven individuals, of which six collected the data, and the seventh was another PHD student from University of Minnesota. The team was divided into subteams of two or three coders, and specific key informant interviews were assigned to the subteams for coding purposes. Each subteam coded a minimum of three interviews. Codebook development was initiated by creating a list of open codes from the key informants’ interview guide, after each member of the team read the interviews. The codes were defined and further redefined during sub-sequent coding cycles. Deductive and inductive codes were added to the final codebook up to the stage of defining categories. Codebook development involved revisions and refinements of the codes and their definitions during all coding steps. Twice a week, subteam meetings were held continuously during the coding process; these meetings involved coding updates, comparison of codes, and reconciliation of any disagreements or coding discrepancies and reaching consensus of coding definitions between coders during the coding process. All team weekly meeting was held for final revision of codebook and verification. This iterative process increased the consistent application of codes to the same portions of text and enhanced the interpretation of findings.

## Results

We interviewed 11 key informants from different professions and backgrounds; these included community leaders, religious leaders, public health experts, cultural experts, and a political leader. Male participants exceeded the number of females (8 males versus 3 females); each informant was asked to give their views about the practices of HCPs when providing sexual health services to patients. Each participant was asked to give his or her opinion based on their own personal experience with HCPs or the feedback they receive from the population that they personally serve, including religious, cultural/tribal members, individual clients, and the community in general.

Following analysis, one main category emerged with three subcategories: the category *Practical aspects to sexual health care* refers to the health care practices when providing sexual health services (Table 1).

### Practical Aspects to Sexual Health Care

Under this category, key informants described how HCPs do their work at a given time when providing sexual health services or any other form of health care. They individually discussed the weakness and strength in the providers’ performances as well as what they see lacking in the services.

#### (i) Strengths during care provision

The participants first acknowledged the availability of some of the sexual health services to patients and then how well sexual health care is provided by HCPs in Tanzania. One informant noted as a strength that services are open to everyone in the society especially public facilities where services are affordable.

“Maybe because the services are open to everyone in the public, at least for public facilities it is cheaper so you can say that is what they are doing good” (public health expert_3_).

Adults were identified as more likely to receive good sexual health services in comparison to children or youth. This is because adults are more mature, are independent, have freedom to choose, or do anything without restrictions from a guardian. Another informant stated that majority of patients in Tanzania receive ethical treatment. They are cared for, valued, and treated well during their recovery. Informants provided a caveat that with the exception of a few health professionals whose practices could be unethical and poor, most health care providers were perceived to be adhering to their codes of conduct and to do their work with commitment, flexibility, and passion.

“I think they are doing a good job dealing with adults, because adults are more understanding and matured with the ability to do, whatever they want in life” (NGO leader_2_).

“Among the things they do best is that they practice “do no harm” code of conduct. So, whether someone comes in with wrong sexual orientation or whatever, the health care provider will handle that person as a human being and address these issues as a human being, as far as the ‘do no harm code’ is concerned and that is good” (public health expert_1_).

“Let me say that most of the health care providers are doing their jobs with a big passion, taking care of people who are naturally considered by the law, community and religion that they are deviants, it is not appropriate to have them in our society but you (health care provider) decided to take care of them it needs passion,… but also flexibility because they (health care providers) provide services day and night even at the times when they are supposed to be with their families” (political leader).

Religious leaders pointed out that apart from providing medical assistance, HCPs play a role of giving advice to patients or clients in need of different health services including sexual related problems. While doing so, the informants mentioned as a strength in health care practice the way some providers observe confidentiality when consulting patients, allowing a room for privacy and comfortability during services.

“I can say they are doing a great job (health care provider), they will advise you what to do for example during pregnancy, not to do heavy work, or sometimes they will tell you it dangerous to be pregnant at this age (young age) you have to wait until you are matured enough and get married. I think this is what they are doing well…” (religious leader_1_).

[General health care service] “I think a patient can speak to a doctor and expect to get information from the doctor and vice versa, this information should not go beyond from these two people and involve other people… and they consider if a patient is comfortable to speak” (religious leader_2_).

#### (ii) Barriers to care provision

One informant mentioned that misconduct is common among HCPs as well in Tanzania. He elaborated on this aspect by saying that this is because most of the health care providers originate from the same communities that they serve, share the same cultural values as the community members, or belong to the same tribe. Therefore, when one is required to act professionally while handling sexual health-related matters, their professional judgment becomes biased. He explained that the provider has the same negative attitude as the broader society towards some sexual related concerns, or they do not have any evidence or understanding of the issues raised by a particular community. Rather, they handle such issues based on their (health care provider’s) own interests. For example, if society members believe that once you teach boys about sex they will become sexually active, some HCPs will support that because they come from the same community (even though sexual education does not lead to promiscuity). Also, some HCP attitudes and decisions may be influenced by their own religious or cultural background. Because of this, they may provide biased care.

“Health care providers are coming from the very society which they are supposed to serve so their attitude, beliefs maybe similar unless there is extra effort imposed to make someone think differently” (cultural expert_1_).

“I think care providers consider themselves as Christians or Africans and decide not to attend patients based on the influence of their own backgrounds” (cultural expert_2_).

Participants raised concerns about the unfriendly policies existing in the country (Tanzania) that contribute to inadequate care of patients especially to populations at highest risk of contracting HIV and other sexually transmitted illnesses, referred to as key populations. These include sex workers, men who have sex with men, and transgender people. They (informants) said that laws and policies discriminate against these vulnerable populations. Providing services to them becomes a huge challenge. In addition, the available treatment guidelines do not support equal provision of health services to such populations, leaving them more at risk.

“I think there is this national policy on treatment of STIs that forces patients to bring their partners for treatment otherwise the index patient may not be treated. It is difficult for sex workers and homosexuals because they may sleep with five or six men per night and if the hospital tells them that they will not be treated unless they bring a partner then how many will they bring?..some bad practices are imposed to the health care providers by the treatment guidelines that denies services to whom we call immoral categories of individuals…this should not make us drag away their(sex workers)rights” (cultural expert_2_).

Furthermore, most key informants reported that a significant proportion of health care professionals are hostile and have negative attitudes towards key populations. They refuse to provide services to them hence accelerating inaccessibility of sexual health services towards these groups, these subpopulations are despised because of the nature of their work and sexual orientation. Additionally, inaccessibility of health services towards key populations is common in public facilities compared to private health facilities as reported by one key informant.

“Those who are negative become very hostile to these patients or clients with uncommon sexual orientation… you find health care providers are not happy or not willing to attend this person and on top of that they use bad words for instance a HIV sex worker went to a clinic and she was refused services and a health care provider as her ‘why should I attend you, you want to live like that so that you can kill others?’…These scenarios exist or even asking for birth control methods becomes a challenge” (public health expert_1_).

“Even homosexuals or sex workers they may access some of those services including STI services, but whenever they go for the services especially in public or government hospital, it becomes difficult to be cared for..they find health care workers being so negative and judgmental” (public health expert_3_).

Key populations were also mentioned as victims of stigma and demeaning judgments from HCPs, due to their sexual orientation and sexual behaviors. Such acts harm patients and eventually teach them to not be open with providers about their real health concerns or worse to avoid health facilities altogether. Another participant stressed about hostility towards younger people, saying that youth unfriendly services have a huge impact on adolescent sexual health. She (informant) explained that most of HCPs are judgmental when giving sexual health services to younger people and stigmatize them.

“I think sexual health services should be provided without stigma. When I am saying that I mean if a young teenage girl comes to the clinic and asks for contraceptives let’s give her a chance to express her concerns to us rather than being too judgmental and having a lot of negative attitudes towards the child” (NGO leader_1_).

“Some health care providers are stigmatizing these key populations a lot or if they give services they do it with judgement because they expect everyone to be a non-key population” (political leader).

#### (iii) Gaps during care provision

The key informants identified gaps in care provision. In their opinion, some health care professionals lack confidentiality in their practices. They may disclose information regarding patients to their colleagues or sometimes to other individuals who are not care providers. This erodes the patient’s trust in the provider.

“Female sex workers and men who have sex with men tell us stories, that whenever they go to hospitals and describe their problems to the care provider for example I have wounds or ulcers around my anus or pus coming out from my penis, vagina or anus, the providers will use bad languages and talk about the patient with their fellows,…so no confidentiality when we real think about passing information to other people” (cultural leader_2_).

Another gap in provision of good care was sufficient time. Participants explained that most HCPs cannot spend enough time with their patients due to being overwhelmed by many patients in their care settings. In Tanzania, there is a very low provider-to-patient ratio. Therefore, HCPs become impatient when giving services to clients. These concerns may create a problem because it limits the client’s freedom to discuss their health concerns with the health care providers.

“Partly because their number is few and they are supposed to rush while providing services and unfortunately these sexual health issues need time to talk with patients” (political leader).

“This is because a doctor has a lot of patients and he or she needs to finish all of them at a specific time… so doctors who are dealing with sexual health issues they have to be patient and give their client enough time to talk” (religious leader_3_).

Lastly, one key informant pointed out that when providing sexual health services, most attention is given to female sexual health care, and not a lot focuses on male concerns or problems. Because men’s sexual health is as important as women’s, efforts should be made to include men in sexual health provision.

“I think we providers we are dealing most with women care when it comes to sexual health and leaving male aside while they need that service as well” (NGO leader_2_).

#### Similarities and Differences Among Key Informants

Across the different types of key informants, the comments were very similar regarding the strengths, barriers, and gaps in provision of care services. With reference to confidentiality, it appeared that informants had diverse opinions. One cultural leader emphasized the lack of confidentiality when (some) HCPs attend clients, while a religious leader emphasized how well health professionals observe confidentiality.

In regard to key populations, most of the respondents focused on how these subpopulations face significant challenges while accessing health care services. However, one religious’ informant of a Muslim background, emphasized that a man who practices anal sex or gay behavior should receive medical services only if he presents with serious symptoms. If symptoms are mild he argued the patient should not receive any sort of treatment because of limited medical resources. Furthermore, he thought the patient should be reported to the police because homosexuality is illegal. His response differed from the Christian leader who advocated to treat all patients regardless of an individual’s sexual orientation. These findings should not be taken to represent the view of their larger religious communities, but the diversity within them.

“If doctors think that there is a possibility for more that 60% that this person will recover, let them treat him just as a human being, but there after they have to counsel him to stop those behaviors .…otherwise they have to report to government for him to be punished… the government has a genuine reason of punishing him, like taking him to jail, it (government) will not let that person spend hospital resources unnecessarily. All I can say is if a gay goes to hospital with problem related to what he is doing he has to be given care if that medication is available but if it is unavailable it’s better to give another person who is not engaging to those behaviors…after treatment they have to report this person because it’s against our laws” (religious leader_1_.

## Discussion

The key informants in this study widely elaborated about the clinical practice of sexual health care provision. From our results, we found that there exist some forms of sexual health care services health facilities in Tanzania, despite the limited efficiency during clinical care provision. However, significant responses explored at the barriers and weaknesses when delivering sexual health services to recipients; these two aspects reflected on the challenges in the clinical practice of the care providers. Furthermore, the findings from this study were important to researchers due to the professional diversity and different backgrounds of the participants involved.

From our study results, public health facilities were regarded as the ones easily accessible and affordable to those seeking health services; this statement was supported by another study that was done in the same context by Shayo et al. (2016). In addition to this, components of sexual health are among the essential health care services provided in both public and non-public health facilities in the country. This allows the availability of some of the sexual health services to the communities, including sexual and reproductive health, HIV/AIDS, and STI services. However, as described in our results, key populations may still be facing challenges in accessing these services and mostly from public facilities. This statement was partly in correspondence with a study done among sex workers in Mozambique, which described perceived barriers towards access of sexual health services from a public health sector, despite the fact that the mentioned public health facilities were the main service providers in comparison to private facilities (Lafort et al., 2016).

Our study findings also show that, there is a significant proportion of HCPs that abide to the ethics of clinical care when treating patients. Such health care providers are passionate and committed and deliver good services including counseling services especially to adult clients. This fact displays how much they value their work as service providers to those in need. It is important to adhere to ethics and professionalism when providing sexual health services, and care must be comprehensive and equal regardless of the recipients’ age differences. In respect to age, our results elaborated about good sexual health care provision to adults in comparison to youth; this idea is supported with various studies (Damian et al., 2020; Fuentes et al., 2018; Mbeba et al., 2012; Muanda et al., 2018; Pandey et al., 2019; Thongmixay et al., 2019) that discuss poor sexual health care provision and the barriers experienced by adolescents and young people while accessing sexual health services compared to adult clients.

In addition to the unfriendly sexual health care provision towards the youth, the stakeholders in our study further discussed other existing barriers that are contributed by the care providers’ practices. An important concern was about the attitude, behavior, moral, or religious values and sociocultural background of the HCP, which may have a negative impact to patients during service provision. Poor providers’ practices correspond with other studies done elsewhere (Duby et al., 2018; Gatuguta et al., 2018; Magesa et al., 2014; Mchome et al., 2015; Wanyenze et al., 2017). Such misconducts may impede patients to disclose sexual health problems to HCPs, hence difficulties in accessing sexual health services and as a result, poor sexual health status among recipients and society in general.

The findings from our study described how the health system may has a role towards inaccessibility of sexual health services through inadequate treatment guidelines and policies, which may result to an increased vulnerability towards sexual-related problems among individuals. Most health conditions have specific guidance that instructs HCPs on the management of patients; these written health policies and guidelines may have medical and legal procedures that are expected to direct HCPs when providing care services. This information is similar to other findings in Africa and globally (Davis et al., 2017; Laar & DeBruin, 2017). Sexual health policies and guidelines should be non-discriminatory, equal and beneficial to all individual regardless of gender, sex, age or orientation. Such policies should be a key guidance to HCPs once faced with sexual health ethical dilemmas during care provision.

Gender equality, time factor, and confidentiality were among the challenges discussed in this study. Our descriptions emphasized on how most of sexual health services are focused on female sexual problems than males’ problems, and this statement is true as seen in some of the local studies (Hokororo et al., 2015; Mbeba et al., 2012; Sigalla et al., 2018). The most probable reason for this could be due to females being quicker in health care seeking than males or some of sexual-related problems are commonly reported by females, for example, sexual and reproductive health issues, female genital mutilation, and gender-based violence and hence more data collected. Moreover, some of the key populations encounter barriers in accessing these services, for instance, men who have sex with men, hence resulting to men becoming a hidden population towards disclosing sexual problems. However, it is conflicted by comparing the few studies done in Africa that have significantly focused on men’s sexual health (Ameh et al., 2012; Botão et al., 2016; Magesa et al., 2014; Mgopa et al., 2017; Pallangyo et al., 2016; Yovwin et al., 2015). Such evidence shows that men’s sexual health problems do exist and interventions of these problems in relation to gender might be helpful in improving the sexual health of men.

Tanzania, and most likely other countries in Sub-Saharan Africa, do struggle with workforce in health care systems (Manzi et al., 2012; Oleribe et al., 2019). The provider-to-patient ratio is very low, in such a way burden of work among health care providers increases, and this may result in time restriction when attending patients. Our study results emphasized about a provider having a lot of patients while attending them during consultation hours; this limits the amount of time scheduled as a standard time for each consultation, and therefore, providers are deemed inefficient during their service provision. Shortage of time forces the provider to be fast, impatient, and in the end failing to explore the details of patients’ or clients’ problems. Time pressure during care provision was also reported in other studies (Jonas et al., 2018; Kingsberg et al., 2019; Mchome et al., 2015). Moreover, we obtained mixed responses about confidentiality; some informants referred to confidentiality and privacy (due to infrastructure) as a strength during sexual health care provision, and others felt that confidentiality was lacking among most health professionals. The latter involved disclosing information about patients to other work and non-work colleagues. Most studies support reports about lack of confidentiality and privacy as a barrier in accessing sexual health services, including a study conducted in Tanzania, which reported this barrier in both ways—as an infrastructural challenge and a breach of information (Mchome et al., 2015). Mixed reactions were also observed among religious leaders, who differed in opinions with regards to sexual care provision towards key populations. This shows that different informants may have opposing views on a similar topic of discussion, but both opinions carry a significant contribution towards improving health care provision.

Our findings have implications to the curriculum development specifically meant for health students in Tanzania and Sub-Saharan Africa at large. Despite the existing clinical practices, our results showed a considerable number of barriers when providing sexual health care and hence the need for developing an Afrocentric sexual health training curriculum. Such training is needed in order to equip future health care providers with clinical knowledge and practical skills regarding sexual health, its related ethics, and policies. As evidenced from recent studies (Abeid et al., 2016; Karimian et al., 2018; Micheni et al., 2017; Renju et al., 2010; Ross et al., 2018), imparting knowledge to health care providers promotes better service provision to patients and confidence to practitioners, thus improving the patient’s sexual health.

### Study Limitations

Given the fact that our study is of a qualitative design and we obtained diverse opinions from eleven key informants from different professional backgrounds, such a sample size may seem small. To complement this in obtaining more generalizable and diverse results in the future, research studies may consider involving actual sexual health service recipients including adolescents and young people and representatives of key populations. The other limitation is that we did not seek opinions from the health care providers who are the ones providing these services to individuals. Capturing their perceptions might have added more insights along with the information obtained from the informants. Despite the limitations encountered, our study provides valuable information which also depended on the profession of the participants, the information provided was partly based on religious, cultural, political, and public health point of view.

### Conclusion and Policy Implication

Our study has identified the strength and barriers of clinical practices related to sexual health. The youth and key populations seem to be much affected by these barriers when accessing sexual health services in comparison to other individuals. Health care providers’ negative attitudes and sociocultural background are contributing to such barriers, as well as poor education, infrastructure, and inadequate policies. It is important for sexual health clinical practices to be guided by professionalism and non-discrimination. Training of HCPs about sexual health care and handling its patients may improve the clinical practice and minimize negative attitudes as well as practical misconducts. Such a training curriculum will be beneficial to HCPs who are in training and in actual clinical practice. Nevertheless, there is a huge need for policy makers at the national level to review existing policies and guidelines as well as developing new policies targeting sexual health care provision in Tanzania. Such policies will have an impact in improving the sexual health needs of the most at-risk individuals.

## Figures and Tables

**Table 1:** Categories, subcategories, and selected codes that emerged during data analysis

Category	Subcategories	Selected codes
Practical aspects of sexual health care	• Strengths during care provision	• Available services
		• Adhere to code of conduct • Good service delivery to adults • Work with passion • Advising and counseling
	• Barriers to care provision	• Provider’s values and background • Unfriendly policies • Relying on wrong guidelines • Youth unfriendly • Stigma • Negative attitude • Judgment • Hostility
	• Gaps during care provision	• Lack of confidentiality • Lack of patience and time • Unavailability of male focused services

## Data Availability

Data is not available as these are key informant interviews that are completely qualitative (not applicable).
